# Effect of Interlayers on Microstructure and Corrosion Resistance of 304/45 Stainless Steel Cladding Plate

**DOI:** 10.3390/ma18112473

**Published:** 2025-05-24

**Authors:** Yongtong Chen, Yi Ding

**Affiliations:** College of Materials Science and Engineering, Nanjing Tech University, Nanjing 210009, China; 202261203128@njtech.edu.cn

**Keywords:** stainless steel cladding plate, carbon diffusion, interlayer, corrosion resistance

## Abstract

During the high-temperature preparation of stainless steel cladding plate, carbon atoms from carbon steel diffused into stainless steel. When temperatures were within 450–850 °C, carbides precipitated at grain boundaries, which initiated intergranular sensitization and thereby reduced the corrosion resistance of stainless steel. This study designed NiP and NiCuP interlayer alloys to effectively block carbon diffusion in stainless steel cladding plates. The effect of adding interlayers on the microstructure of stainless steel cladding plate was studied by using optical microscopy and scanning electron microscopy. Electrochemical tests were subsequently conducted to evaluate the impact of interlayer incorporation on the corrosion resistance of stainless steel cladding. The results demonstrated that 304/45 specimens exhibited severe carbon diffusion, resulting in the poorest corrosion resistance. The addition of interlayers improved the corrosion resistance of stainless steel cladding to varying degrees. Among these, the 304/NiCuP/45 specimen showed the best performance. It had an intergranular corrosion susceptibility of only 0.25% and pitting potential as high as 0.336 V, which indicated its superior corrosion resistance. The passive film of stainless steel cladding exhibited n-type semiconductor characteristics. And 304/NiCuP/45 specimen demonstrated the lowest carrier density of 3.02 × 10^18^ cm^−3^, which indicated the formation of the densest passive film.

## 1. Introduction

Stainless steel cladding plate (SSCP) represents an advanced composite material that strategically combines stainless steel (SS) and carbon steel (CS) through metallurgical bonding [1,2]. SSCP has been extensively used in petroleum engineering, marine engineering, metallurgical industries, and power systems owing to its synergistic combination of excellent corrosion resistance from SS and superior mechanical properties contributed by CS [3,4].

At present, the production and processing methods for SSCP include explosive welding, hot-rolling composite, and casting composite [5]. However, due to differences in material composition between SS and CS, carbon elements diffuse from CS to SS during the composite process, forming carbide precipitates [6]. The impact of carbide precipitation on microstructure and properties of SSCP has long been a key focus in research on this material [7]. Tao et al. [8] discovered that sensitized zones composed of grooves and Cr-depleted zones appeared on the SS side, with the precipitates identified as Cr-rich carbides. Jiang et al. [9] investigated the effects of heat treatment on the microstructure and mechanical properties of Ti-steel composite plates. Their study revealed that heat treatment promotes the interdiffusion of interfacial elements and induces microstructural transformations. Specifically, carbon diffusion into SS combines with chromium to form carbide precipitates, which leads to chromium depletion at SS grain boundaries and a subsequent decline in the corrosion resistance of SS cladding [10]. Thus, adding an interlayer in SSCP preparation to block carbon diffusion and enhance their corrosion resistance was highly significant for actual production [11]. However, an improper interlayer choice might instead degrade the properties of SSCP.

Wang et al. [12] investigated the addition of Fe interlayers had minimal impact on interfacial elemental diffusion, while the incorporation of Ni and Nb interlayers effectively suppressed carbon diffusion behavior at the interface. Xiao et al. [13] investigated that the Q235B side with Ni interlayers showed no apparent decarburization layer, while distinct decarburization layers formed in specimens with Fe and Nb interlayers. Xie et al. [14] revealed that Nb interlayers effectively suppressed interdiffusion among Ti, C, and Fe elements at 800 °C. However, when the heat treatment temperature increased to 900 °C and 1000 °C, the formation of Nb-Ti solid solutions at the bonding interface caused significant deterioration in mechanical performance. Yu et al. [15] found that pure Fe interlayers effectively prevented carbon migration from CS to SS, thereby inhibiting the formation of Cr-carbides and enabling the composite plates to achieve superior mechanical performance. Yang et al. [16] findings demonstrated that Ni interlayers lost their inhibitory efficacy on Ti and Fe interdiffusion when the temperature reached 1000 °C, leading to extensive formation of brittle TiC and TiFe intermetallics at the interface, which consequently caused a significant reduction in shear strength. Lin et al. [17] revealed that Ni interlayer can effectively block carbon diffusion while enabling robust metallurgical bonding between CS and SS, thereby significantly enhancing the mechanical properties of SSCP.

In summary, the incorporation of an interlayer with appropriate wettability into SSCP could effectively block carbon diffusion. Current research findings predominantly focus on the influence of interlayer addition on carbon diffusion and mechanical properties of SSCP [18]. While carbon diffusion played a decisive role in the corrosion resistance of SSCP, studies investigating the impact of carbon-blocking interlayers on the corrosion resistance of SS cladding remained relatively limited. Given that nickel-based alloys could incorporate a large number of other alloying elements and still maintain basic corrosion resistance, the interlayer alloy in this study was based on nickel-based alloys. Cu, a commonly used brazing material for steel, exhibits good wettability with both CS and SS, while P reduces the melting point of the interlayer alloy. Accordingly, this study employed 304 SS and 45 CS as base materials, with NiP and NiCuP alloys as interlayers, to investigate the effects of interlayer addition on the microstructure and corrosion resistance of SSCP. Particular attention was devoted to the impact of the interlayer on carbon diffusion in SSCP and the corrosion resistance of SS cladding.

## 2. Materials and Methods

### 2.1. Materials and Preparation

SSCP fabricated in this study used 304 stainless steel (Runqiao, Fujian, China) and 45 carbon steel (Runqiao, Fujian, China) as base materials, with their chemical compositions listed in Table 1. Interlayer alloys selected in this study were NiP and NiCuP alloys, and their specific chemical compositions were provided by the manufacturer and calculated as shown in Table 2. A set of SSCP without an interlayer was selected for microstructure and properties comparison.

The prepared interlayer slurry was evenly spread on the surface of an 18 mm thick CS plate, then covered with a 2 mm thick SS plate, resulting in an original slab with a total thickness of approximately 20 mm. The original slab was held at 1050 °C for 60 min and then subjected to rolling bonding through six passes, achieving a total reduction of 15 mm. The final SSCP achieved a thickness of 5 mm, the thickness of the SS cladding was 0.5 mm, and the thickness of the interlayer was 0.05 mm.

### 2.2. Microstructural Characterization

First, specimens with dimensions of 10 mm × 10 mm were cut from SSCP and mounted in epoxy resin. The specimens were progressively ground using silicon carbide sandpapers with grits of 120#, 320#, 600#, 1200#, and up to 2000#, followed by polishing treatment. Finally, the specimen surfaces were cleaned with ethanol. Corrosion of the SS side was achieved with 10% oxalic acid at 7 V for 20 s, and the CS side was corroded with 4 vol.% nital until surface color changes were observed. The examination of the microstructure was conducted with an optical microscope (OM) sourced from Oberkochen, Germany. Additionally, the surface morphology along with elemental composition was analyzed through scanning electron microscopy (SEM, model SU5000) coupled with energy-dispersive X-ray spectroscopy (EDS) from Oxford, UK.

### 2.3. Mechanical Performance

The hardness of SSCP was measured using an HVT-1000 fully automatic micro Vickers hardness tester with a test load of 100 gf. Taking the bonding interface between SS and interlayer as the 0 μm reference point, gradient measurements were performed perpendicular to the interface toward both the SS side and CS side, with a spacing of 50 μm between each measurement point. All data were presented as the average of three independent measurements.

### 2.4. Electrochemical Corrosion Properties

To evaluate the influence of incorporating an interlayer on the corrosion resistance of SSCP, electrochemical tests were conducted after reducing the SS cladding layer thickness to 50 μm through polishing. Specimens were sanded and polished with sandpaper after being created as electrochemical specimens using epoxy resin. The CHI760E electrochemical workstation was thereafter used for all electrochemical experiments.

After the specimens were held in an open circuit for one hour, electrochemical impedance spectroscopy (EIS), Mott–Schottky, and potentiodynamic polarization tests were conducted, respectively. This study investigated the corrosion resistance of SSCP in a simulated seawater environment and a 3.5 wt.% NaCl solution was selected as the experimental solution. EIS measurements were performed over a frequency range of 10^5^ Hz~10^−2^ Hz with an amplitude of 10 mV. Potentiodynamic polarization scans were conducted from −400 mV to +400 mV (vs OCP) at a scan rate of 1.5 mV/s. For Mott–Schottky tests, the potential was scanned from −400 mV to +200 mV (vs OCP) with a 50 mV amplitude and a fixed frequency of 1000 Hz.

The DL-EPR method can evaluate the degree of sensitization (DOS) of SS cladding, calculated as the ratio of reactivation current (I_r_) to activation current (I_a_). The test solution consisted of 0.5 mol/L H_2_SO_4_ + 0.01 mol/L KSCN, with a potential scan rate of 1.5 mV/s. After scanning to +0.2 V, the potential was reversed at the same rate back to open-circuit potential. Upon completion of the electrochemical assessment, corrosion morphology was examined using scanning electron microscopy.

## 3. Results and Discussion

### 3.1. Microstructure Characterization

**Figure 1:**
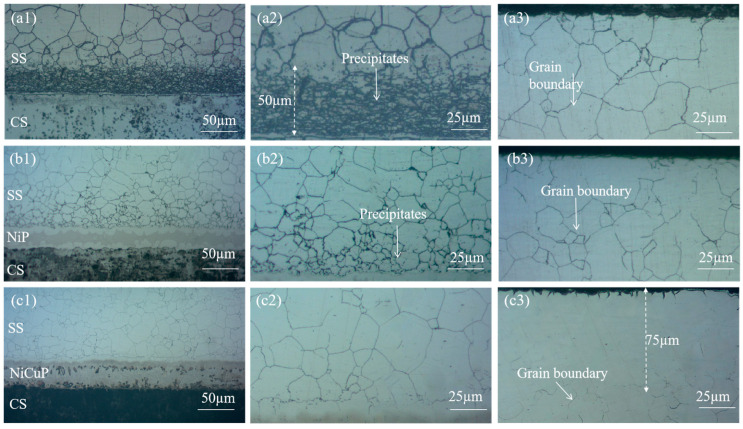
Metallographic micrographs of SSCP: bonding interface (**a1**) 304/45, (**b1**) 304/NiP/45, (**c1**) 304/NiCuP/45; near-interface region of SS cladding (**a2**) 304/45, (**b2**) 304/NiP/45, (**c2**) 304/NiCuP/45; surface region of SS cladding (**a3**) 304/45, (**b3**) 304/NiP/45, (**c3**) 304/NiCuP/45.

Figure 1 shows the metallographic microstructure of SSCP. The microstructure of the 304/45 specimen was shown in Figure 1(a1–a3), where a distinct carburized layer with a network structure was observed throughout the SS cladding, accompanied by coarse grain boundaries. A 50 μm black precipitate layer was visible near the bonding interface of the SS cladding. Meanwhile, severe decarburization was observed on the CS side. These phenomena indicated significant C diffusion had occurred in the 304/45 specimen, with sufficient C diffusion reaching the SS cladding surface [19]. The microstructure of the 304/NiP/45 specimen is depicted in Figure 1(b1–b3). A network carburized layer was also observed throughout the SS cladding, but the amount of black precipitates was substantially reduced compared to the 304/45 specimen. Only minor decarburization was detected on the CS side. This was attributed to Ni, a non-carbide-forming element, which reduced the diffusion coefficient of C, thereby effectively slowing the diffusion of C within the interlayer and partially blocking C diffusion, though not completely [20]. The microstructure of the 304/NiCuP/45 specimen is presented in Figure 1(c1–c3). A carburized layer was observed near the bonding interface of the SS cladding, while no network grain boundaries were detected within a region approximately 75 μm from the SS cladding surface. Additionally, no decarburization was observed on the CS side. This demonstrates that the addition of NiCuP interlayer had blocked most C diffusion from the CS side to the SS side, allowing only a small amount of C to diffuse into the SS cladding without reaching its surface [21].

**Figure 2:**
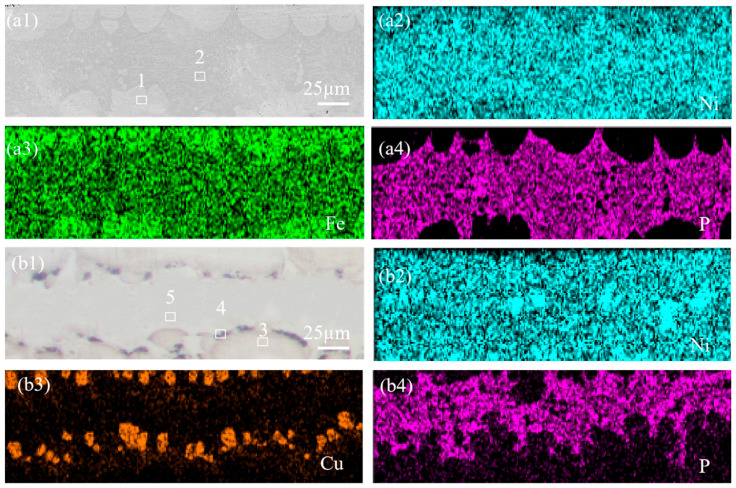
SEM images and corresponding elemental distributions of interlayers: (**a1**) NiP interlayer; corresponding EDS elemental mappings of (**a2**) Ni; (**a3**) Fe; (**a4**) P; (**a1**) NiCuP interlayer; corresponding EDS elemental mappings of (**b2**) Ni; (**b3**) Cu; (**b4**) P.

The interlayer undergoes interdiffusion of elements with both bonded metals during the preparation process, and elemental segregation might occur during solidification, leading to compositional changes in the interlayer. Therefore, SEM and EDS analysis were employed to characterize the microstructure of NiP and NiCuP interlayers. The EDS results in Figure 2 are listed in Table 3. Figure 2(a1) presents the SEM image of the NiP interlayer, revealing wave-like white solid solution phases and central eutectic structures. EDS results indicated that the white solid solution (1#) primarily consists of Ni (53.91 ± 0.54%) and Fe (40.32 ± 1.58%), with P content below 1%. This was attributed to Fe diffusion into the interlayer driven by concentration gradients during the preparation process. During solidification, the high-melting-point Fe-Ni phases preferentially precipitated at the interface [22]. In contrast, the central eutectic structure (2#) was rich in P (14.96 ± 3.3%), forming a Ni-Fe-P eutectic phase. Figure 2(b1) shows the SEM image of the NiCuP interlayer, featuring wave-like light-red solid solution phases, elliptical dark-red regions, and central eutectic structures. EDS analysis identified the light-red wave-like solid solution (3#) as a Fe-Ni-Cu solid solution, the elliptical dark-red regions (4#) as Cu-rich zones (up to 45.43 ± 0.79% Cu), and the central eutectic structure (5#) as a Ni-Fe-P eutectic phase.

**Figure 3:**
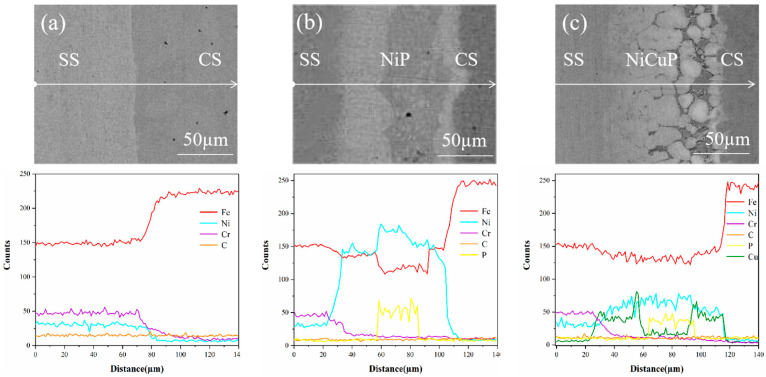
Line scanning profiles near the bonding interface of SSCP: (**a**) 304/45; (**b**) 304/NiP/45; (**c**) 304/NiCuP/45.

During the preparation process of SSCP, elemental interdiffusion occurred. Line scanning characterization of the bonding interface is shown in Figure 3. In the 304/45 specimen, Ni and Cr diffused from SS to CS, while Fe diffused from CS to SS. In the 304/NiP/45 specimen, Ni diffused from the interlayer to both bonded metals, and Cr diffused from SS through the entire interlayer into CS. Fe in CS diffused into the interlayer, while Fe from SS was also observed to migrate toward the interlayer, and Fe enrichment was observed at the interface on both sides of the interlayer. Notably, no diffusion of P into either bonded metal was detected. For the 304/NiCuP/45 specimen, both Ni and Cu diffused from the interlayer to the bonded metals, while Cr diffused from the SS into the interlayer and further into CS. Concurrently, Cu enrichment was observed at both interlayer interfaces. Similarly, no diffusion of P into the bonded metals occurred. As a non-carbide-forming element, Cu effectively blocked C diffusion. The enrichment of Cu at interlayer interfaces created two barrier layers, which may explain the superior C-blocking performance of the NiCuP interlayer compared to the NiP interlayer.

**Figure 4:**
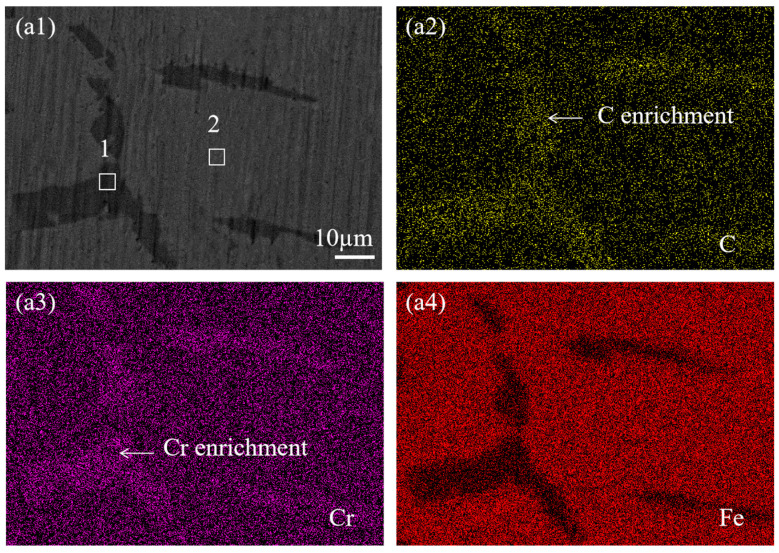
SEM images and corresponding elemental mapping of grain boundaries in SS cladding of 304/45 SSCP: (**a1**) 304/45; corresponding EDS elemental mappings of (**a2**) C; (**a3**) Cr; (**a4**) Fe.

To investigate the elemental composition of precipitates in SS cladding of SSCP, mapping was used to analyze the elemental distribution at the grain boundaries of SS cladding. Figure 4 shows the elemental mapping results of the grain boundaries in the SS cladding of the 304/45 specimen. Figure 4(a1) showed coarse-grain boundaries, which indicated substantial precipitation and enrichment of precipitates along the grain boundaries. From Figure 4(a2–a4), inhomogeneous distributions of C, Cr, and Fe across the grain boundaries were evident. Specifically, C and Cr were enriched at the grain boundaries, while Fe exhibited depletion in these regions.

**Figure 5:**
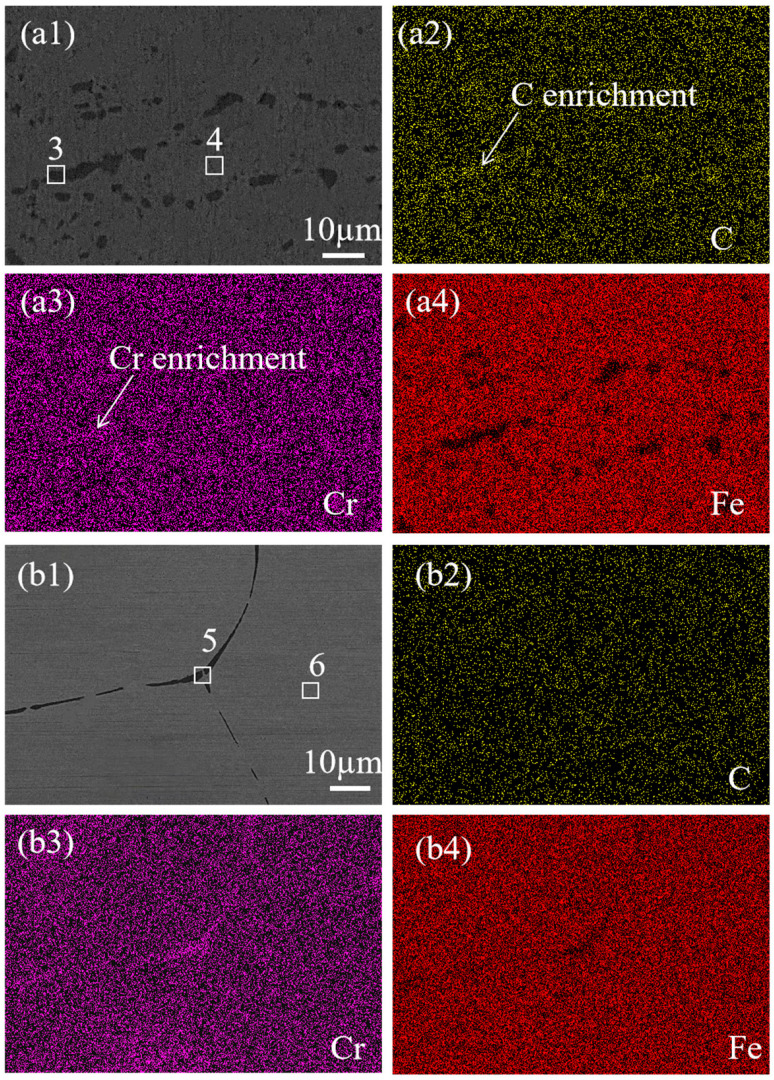
SEM images and corresponding elemental mapping of grain boundaries in SS cladding of SSCP: (**a1**) 304/NiP/45; corresponding EDS elemental mappings of (**a2**) C; (**a3**) Cr; (**a4**) Fe; (**b1**) 304SS/NiCuP/45; corresponding EDS elemental mappings of (**b2**) C; (**b3**) Cr; (**b4**) Fe.

The mapping results of the SS cladding grain boundaries of the 304/NiP/45 specimen are shown in Figure 5(a1). Precipitates were mainly concentrated on the grain boundaries, but the significant reduction in grain boundary width indicated a substantial decrease in precipitate formation. Further observation of Figure 5(a2–a4) revealed clear enrichment of C and Cr at the grain boundaries, while Fe exhibited depletion in these regions. For the 304/NiCuP/45 specimen, Figure 5(b1) displays the elemental mapping of SS cladding, showing clean grain boundaries with further reduced width. From the elemental distribution in Figure 5(b2–b4), C was uniformly distributed across and around the grain boundaries without enrichment or depletion. Although Cr showed slight enrichment at the grain boundaries, the extent was relatively minor. Similarly, Fe depletion at the grain boundaries was observed, but to a lesser degree compared to the 304/NiP/45 specimen.

The elemental composition of the marked precipitates in SEM images was analyzed by EDS, the results of which are listed in Table 4. In the 304/45 specimen, grain boundary regions (1#) exhibited substantially higher chromium (66.7 ± 0.9%) contents compared to grain interior regions (2#), demonstrating that precipitates were predominantly Cr-rich carbides. Similar elemental segregation patterns were observed in the 304/NiP/45 specimens, where grain boundary precipitates (3#) maintained elevated Cr levels relative to adjacent grain interiors (4#). In contrast, the 304/NiCuP/45 specimen showed only minimal chromium enrichment observed at boundary regions. The intergranular corrosion susceptibility of austenitic SS fundamentally arises from carbide precipitation along grain boundaries and subsequent Cr-depleted zone formation [23]. Thus, combined with the above EDS analysis results, it can be concluded that precipitates in SS cladding were mainly Cr-rich carbides, which preferentially precipitate along the grain boundaries.

### 3.2. Mechanical Properties

**Figure 6:**
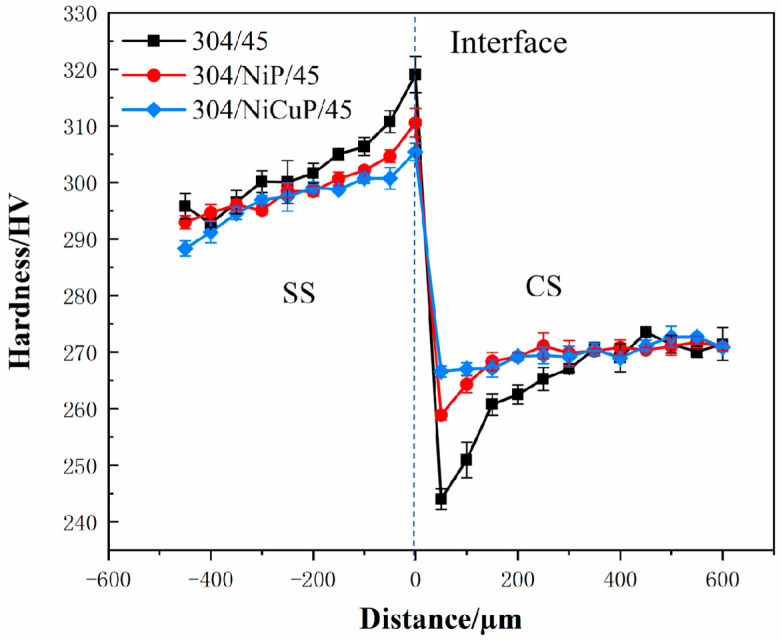
Hardness distribution of SSCP.

Figure 6 shows the hardness test results of SSCP. Hardness gradually increased from the SS cladding surface to the bonding interface. The highest hardness was observed in the near-interface region of the SS cladding in the 304/45 specimen, which was primarily attributed to significant C diffusion from CS to SS cladding, forming carbide precipitates that induced dispersion strengthening [24]. In contrast, the 304/NiCuP/45 specimen exhibited the lowest hardness in the SS near-interface region, which was primarily due to NiCuP interlayer blocking most C diffusion, resulting in minimal carbide precipitation.

On the CS side, hardness increases gradually and then stabilizes. The near-interface region of CS exhibited lower hardness than other CS regions due to decarburization caused by C diffusion, where pearlite completely decomposed into ferrite [25]. The addition of interlayers improved the hardness of the CS near-interface region compared to the 304/45 specimen by suppressing C diffusion. In the CS core region, where no C diffusion occurs, hardness remains stable.

### 3.3. Electrochemical Testing

**Figure 7:**
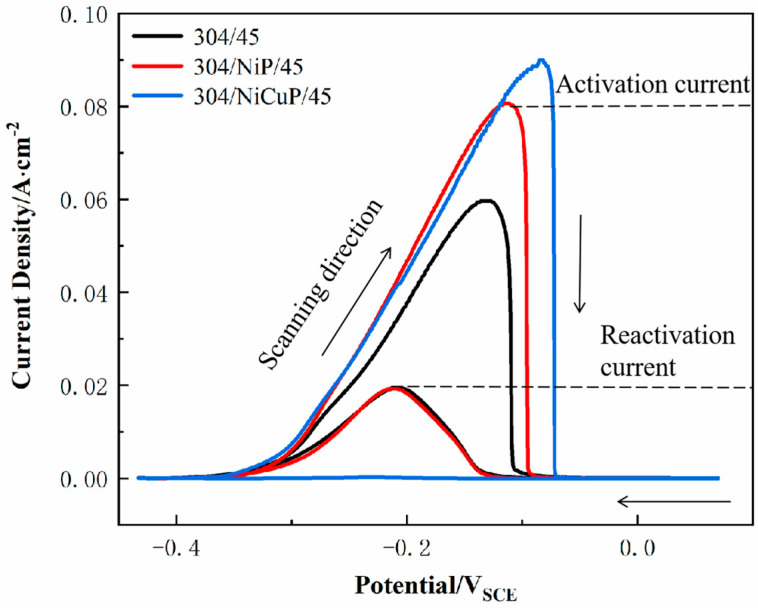
DL-EPR curves of SS cladding in SSCP.

The DL-EPR test was used to evaluate a DOS of SS cladding, aiming to investigate the effect of interlayer addition on the intergranular corrosion resistance of SS cladding in SSCP, as shown in Figure 7. The electrochemical parameters obtained according to DL-EPR tests were summarized in Table 5. The results indicated that 304/45 specimens exhibited an activation current density (I_a_) of 6.27 × 10^−2^ A/cm^2^ during the forward scan and a reverse current density (I_r_) of 2.04 × 10^−2^ A/cm^2^ during the reverse scan, resulting in a DOS of 32.5%. The highest DOS value indicated severe C diffusion in the 304/45 specimen, where extensive Cr-rich carbide precipitation at grain boundaries led to Cr-depleted zones adjacent to boundaries. During the reverse scan in the reactivation region, these Cr-depleted zones, which lacked protective passive films, underwent preferential corrosion, which elevated I_r_ and DOS [26]. In contrast, the 304/NiP/45 specimen showed a DOS of 24.1%, significantly lower than 304/45. However, the reactivation current I_r_ remained relatively high, indicating substantial degradation of Cr-depleted zones during reverse scanning and incomplete passivation film formation [27]. This suggested that while the NiP interlayer partially blocked C diffusion, significant C migration still occurred. For the 304/NiCuP/45 specimen, the reactivation current during the reverse scan was negligible, with no discernible reactivation peak in the curve, yielding an extremely low DOS of 0.25%. This minimal DOS confirmed that the NiCuP interlayer effectively suppressed C diffusion, preventing chromium depletion and enabling the formation of a continuous passivation film on SS cladding [28]. Consequently, the 304/NiCuP/45 specimen exhibited optimal intergranular corrosion resistance.

**Figure 8:**
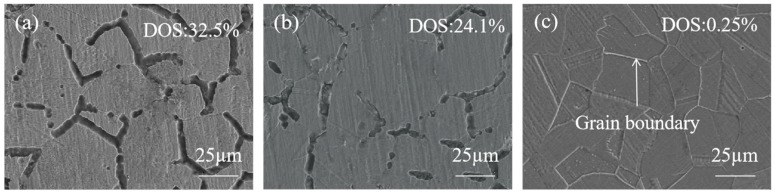
Corrosion morphology of SS cladding after DL-EPR test: (**a**) 304/45; (**b**) 304/NiP/45; (**c**) 304/NiCuP/45.

As can be seen in Figure 8, the corrosion morphology of specimens was shown after DL-EPR tests. The difference in corrosion morphology under SEM was basically consistent with the difference in DOS values. As observed in Figure 8a, numerous holes were present at grain boundaries, forming a network trench structure. This phenomenon was attributed to 304/45 SSCP exhibiting a high DOS of 32.5%, which indicated severe intergranular sensitization. The corrosion of carbides at grain boundaries generated abundant holes, which subsequently coalesced into the observed trench network. Figure 8b revealed that 304/NiP/45 SSCP also exhibited numerous holes at grain boundaries, though only localized regions developed network trenches, with its DOS reduced to 24.1%. In contrast, Figure 8c demonstrated well-defined grain boundaries in 304/NiCuP/45 SSCP, where the DOS was significantly lowered to 0.25%, showing negligible intergranular corrosion.

**Figure 9:**
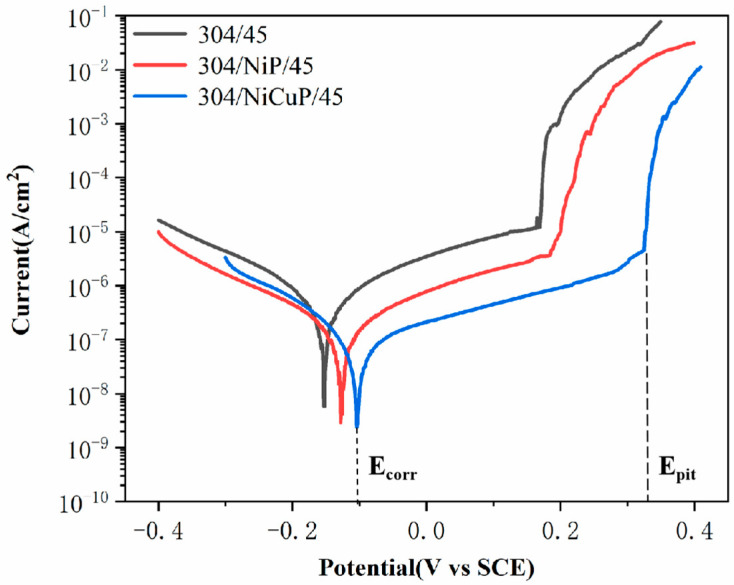
Potentiodynamic polarization curves of SS cladding in 3.5 wt.% NaCl solution.

Figure 9 shows the potentiodynamic polarization curves of SS cladding in SSCP immersed in 3.5 wt.% NaCl solution. Table 6 lists the electrochemical parameters, including corrosion potential (E_corr_), corrosion current density (I_corr_), and pitting potential (E_pit_). The results demonstrated that 304/45 specimens exhibited the lowest E_pit_ (0.178 V) and the highest I_corr_(0.605 μA/cm^2^), attributed to severe C diffusion in SSCP. Cr was the primary constituent of the passive film in SS cladding. The precipitation of Cr-rich carbides induced the formation of Cr-depleted zones adjacent to grain boundaries, thereby compromising the integrity of the passive film and resulting in diminished corrosion resistance [29]. In contrast, the 304/NiP/45 specimen showed a higher E_pit_ (0.196 V) and lower I_corr_ (0.212 μA/cm^2^). This was attributed to the NiP interlayer, which effectively blocked C diffusion, thereby partially suppressing the precipitation of Cr-rich carbides. Further comparative analysis revealed that the 304/NiCuP/45 specimen exhibited the highest E_pit_ (0.336 V) and the lowest I_corr_ (0.086 μA/cm^2^). This was attributed to the NiCuP interlayer effectively blocking C diffusion, which suppressed Cr-rich carbide precipitation and thereby inhibited the formation of Cr-depleted zones [30]. As a result, a continuous and dense passive film formed on the SS cladding, which effectively blocked the intrusion of corrosive species.

**Figure 10:**
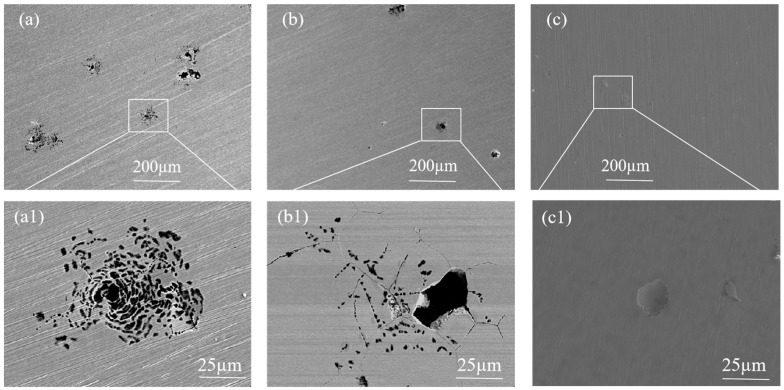
Corrosion morphology of stainless steel cladding after potentiodynamic polarization testing in 3.5 wt.% NaCl solutions: (**a**) 304/45; (**b**) 304/NiP/45; (**c**) 304/NiCuP/45; Pitting pit magnification: (**a1**) 304/45; (**b1**) 304/NiP/45; (**c1**) 304/NiCuP/45.

Figure 10 shows the corrosion morphology of SS cladding after potentiodynamic polarization test in 3.5 wt.% NaCl solution. As shown in Figure 10a, honeycomb-shaped pits surrounded by numerous smaller pits were observed on the SS cladding surface of the 304/45 specimen. Next, EDS spectral analysis was conducted on the uncorroded location 1# at the edge of the corrosion pit. High contents of Fe (58.8 ± 1.12%), Cr (18.78 ± 1.83%), and O (8.16 ± 0.23%) were detected at location 1#. At location 2# inside the corrosion pit, up to 83.76% Fe element was detected, along with a small amount of Cr element (4.51 ± 0.38%), and Cl element (5.13 ± 0.69%) was additionally detected. Compared to location 1#, the Cr element content inside the corrosion pit significantly decreased, while the Fe element content significantly increased. This indicated that Cl^−^ in solution first attacked the defective sites of the passive film, then penetrated the passive film to come into contact with the metal substrate, thereby triggering pitting corrosion. Figure 10b presents the corrosion morphology of the 304/NiP/45 specimen, where severe pitting corrosion was also evident on the SS cladding surface. Figure 10c illustrates the corrosion morphology of the SS cladding in the 304/NiCuP/45 specimen. As shown in the figure, the surface of the SS cladding exhibited virtually no visible corrosion pits. This observation further confirmed that the 304/NiCuP/45 specimen exhibited the optimal corrosion resistance in 3.5 wt.% NaCl solution environment.

**Figure 11:**
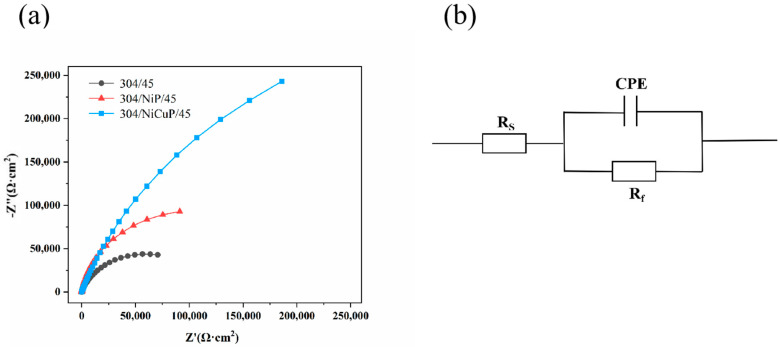
EIS of stainless steel cladding in 3.5 wt.% NaCl solution: (**a**) Nyquist plot; (**b**) Equivalent circuit diagram.

EIS results of SS cladding in 3.5 wt.% NaCl solution is shown in Figure 11. The Nyquist plots were fitted using the equivalent circuit illustrated in Figure 11b, which consists of three components: the combined solution resistance (R_s_) of electrode and electrolyte, double layer capacitance (CPE), and charge transfer resistance (R_f_) at the electrode/electrolyte interface. The fitting results of these parameters are summarized in Table 7. As observed in Figure 11a, all specimens exhibited a single semicircular capacitive arc. A larger capacitive arc radius corresponded to a denser passive film on the material surface. R_f_, which reflected the kinetics of electrode reactions, was inversely related to corrosion rate−higher R_f_ values indicated better corrosion resistance [31]. The results revealed that 304/45 specimens have the smallest capacitive arc radius and the lowest R_f_ (1.19 × 10^5^ Ω·cm^2^), indicating the incomplete passive film and the poorest corrosion resistance. In contrast, specimens with interlayers showed increased capacitive arc radius and R_f_ values, suggesting improved passive film integrity as carbide precipitation decreases. 304/NiCuP/45 specimen exhibited the largest capacitive arc radius and the highest R_f_ (8.51 × 10^5^ Ω·cm^2^). This maximum R_f_ signifies the strongest inhibition of charge transfer at the electrode/electrolyte interface, resulting in the slowest corrosion rate and optimal corrosion resistance.

**Figure 12:**
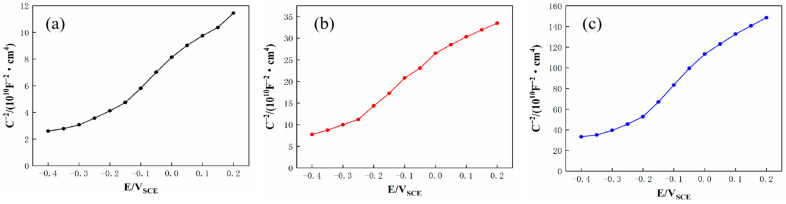
Mott–Schottky curves of stainless steel cladding in 3.5 wt.% NaCl solution: (**a**) 304/45; (**b**) 304/NiP/45; (**c**) 304/NiCuP/45.

The passive film formed on SS cladding in 3.5 wt.% NaCl solution typically exhibited semiconductor properties, and the semiconducting characteristics of this passive film directly influence the corrosion resistance of SS cladding [32]. These semiconductor properties could be evaluated by using Mott–Schottky curves. Figure 12 shows the M−S curves of SS cladding in 3.5 wt.% NaCl solution, with calculated carrier density (N_D_) values derived from Figure 12 listed in Table 8. The figure reveals that within the potential range of −0.4 to 0.2 V, the positive slope of the curve indicates n−type semiconductor behavior in the passive film of SS cladding. According to the point defect model, oxygen vacancies dominate as the primary defect in n−type semiconducting passive films. When such passive films were exposed to Cl^−^—containing environments, oxygen vacancies reacted with Cl^−^ ions to generate new oxygen vacancies. These newly formed vacancies further react with Cl^−^, creating a self-accelerating cycle of vacancy generation. The accumulation of oxygen vacancies introduces structural defects that degrade the integrity of the passive film at the SS surface. As shown in Table 8, the 304/45 specimen exhibits the highest N_D_ (46.85 × 10^18^ cm^−3^) in its SS cladding, indicating that the passive film formed in 3.5 wt.% NaCl solution contains the highest concentration of defects. Specimens with interlayers showed reduced N_D_ values to varying degrees. Notably, the 304/NiCuP/45 specimen achieved the lowest N_D_ (3.02 × 10^18^ cm^−3^), signifying the slowest charge transfer kinetics at the SS surface and formation of the passive film with minimal defects and optimal density in 3.5 wt.% NaCl solution [33]. Mott–Schottky test results aligned with conclusions derived from EIS analysis.

**Figure 13:**
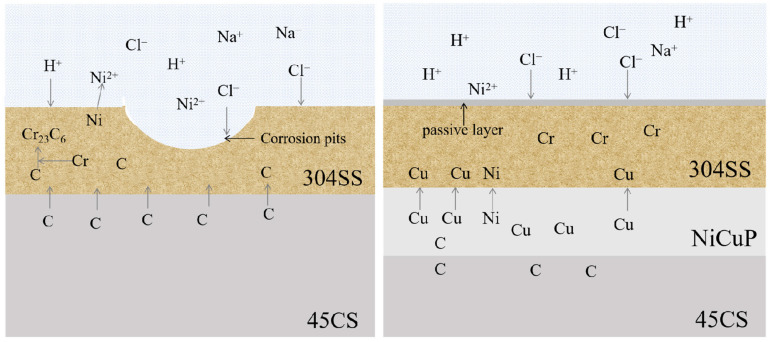
Schematic diagram illustrating the corrosion behavior of SS cladding in 3.5 wt.% NaCl solution.

Figure 13 shows a schematic diagram of corrosion of the SS side of SSCP with added NiCuP interlayer in 3.5 wt.% NaCl solution. The passive film of SS consists mainly of Cr_2_O_3_ [34]. In the case of 304/45, substantial carbon diffusion from the CS side to the SS side led to the formation of Cr_23_C_6_ carbides through chromium segregation [35]. This produced a large number of Cr-depleted zones along the grain boundaries of SS, which resulted in SS cladding not forming a complete passive film. Consequently, Cl^−^ could invade the passive film on the surface of SS cladding and corrode at defective locations on the film surface, which forms pitting pits in 3.5 wt.% NaCl solution. After the addition of the NiCuP interlayer, since Cu was a non-carbide forming element and enriched on both sides of the interlayer, the NiCuP interlayer blocked the diffusion of most C atoms. As a result, SS cladding hardly formed any Cr-depleted zones and could still develop a complete and dense passive film, resulting in significantly enhanced corrosion resistance.

## 4. Conclusions

(1) Different degrees of intergranular sensitization were observed in SS cladding of 304/45 and 304/NiP/45 specimens, with the most severe intergranular sensitization in 304/45. In contrast, almost no intergranular sensitization occurred in 304/NiCuP/45, which was attributed to the fact that the NiCuP interlayer blocked the diffusion of most of the C atoms;
(2) The DOS value of 304/45 was 32.5%, and then the DOS value of 304/NiP/45 decreased compared to 304/45. While 304/NiCuP/45 had the smallest DOS of 0.25%, SS cladding had the lowest intergranular sensitization. Because Cu was a non-carbide-forming element, the addition of NiCuP interlayer effectively blocked the diffusion of C atoms and reduced carbide precipitation at grain boundaries, thus improving intergranular corrosion resistance;
(3) 304/NiCuP/45 SS cladding had the lowest I_corr_ (0.086 μA/cm^2^), and the most E_pit_ (0.336 V), which provided excellent pitting resistance. This was attributed to the fact that SS cladding almost did not produce a Cr-depleted zone, the Cr element was uniformly distributed, and a complete passive film could be formed;
(4) The passive film of SS cladding exhibited n-type semiconductor characteristics. 304/45 specimen exhibited the highest N_D_, while the SS cladding with interlayer showed varying degrees of reduction in carrier density. Among them, the 304/NiCuP/45 specimen demonstrates the lowest N_D_ of 3.02 × 10^18^ cm^−3^, corresponding to the fewest defects in the passive film.

## Figures and Tables

**Table 1:** The chemical compositions of SS and CS (wt.%).

Materials	C	Mn	P	Ni	Cr	Fe
304 SS	0.055	1.21	0.028	8.13	18.28	Bal.
45 CS	0.43	0.55	0.013	0.16	0.21	Bal.

**Table 2:** Chemical composition of two interlayers (wt.%).

Interlayer	Ni	Cu	P
NiP	89	/	11
NiCuP	53.4	36.8	9.8

**Table 3:** The EDS analysis results of different regions in Figure 2

Element	Ni (at%)	Cu (at%)	Fe (at%)	P (at%)
1#	53.91 ± 0.54	/	40.32 ± 1.58	1.51 ± 0.04
2#	52.32 ± 0.9	/	30.53 ± 1.48	14.96 ± 3.3
3#	23.54 ± 1.12	18.95 ± 0.47	51.64 ± 1.18	1.05 ± 0.3
4#	16.92 ± 0.54	45.43 ± 0.79	33.72 ± 1.12	0.75 ± 0.45
5#	20.97 ± 0.81	1.34 ± 0.27	41.63 ± 1.79	23.7 ± 2.1

**Table 4:** EDS analysis results of different regions in SEM images.

Element	Cr (at%)	Fe (at%)
1#	66.7 ± 0.9	33.4 ± 1
2#	30.9 ± 0.6	69.1 ± 0.6
3#	59.5 ± 1.8	40.5 ± 1.8
4#	26.3 ± 1.5	73.7 ± 1.5
5#	37.8 ± 0.8	62.2 ± 0.8
6#	25.3 ± 1.2	74.8 ± 1.2

**Table 5:** DOS values obtained from DL-EPR curves of SS cladding.

Specimen	I_a_ (mA/cm^2^)	I_r_ (mA/cm^2^)	DOS (%)
304/45	6.27 × 10^−2^	2.04 × 10^−2^	32.5
304/NiP/45	8.18 × 10^−2^	2.03 × 10^−2^	24.1
304/NiCuP/45	9.00 × 10^−2^	2.25 × 10^−4^	0.25

**Table 6:** Electrochemical corrosion data extracted from the potentiodynamic polarization curves.

Specimen	E_corr_(V)	I_corr_(μA/cm^2^)	E_pit_(V)
304/45	–0.153	0.605	0.178
304/NiP/45	–0.128	0.212	0.196
304/NiCuP/45	–0.104	0.086	0.336

**Table 7:** EIS fitting parameters of stainless steel cladding in 3.5 wt.% NaCl solution.

Specimen	R_s_ (Ω·cm^2^)	R_f_ (Ω·cm^2^)	CPE (S·sec^n^·cm^−2^)	n
304/45	7.441	1.19 × 10^5^	5.35 × 10^−5^	0.8065
304/NiP/45	8.214	2.14 × 10^5^	6.03 × 10^−5^	0.9186
304/NiCuP/45	4.264	8.51 × 10^5^	2.21 × 10^−5^	0.8055

**Table 8:** Carrier density of the passive film on stainless steel coating in 3.5 wt.% NaCl solution.

Specimen	N_D_ (×10^18^·cm^−3^)
304/45	46.85
304/NiP/45	15.62
304/NiCuP/45	3.02

## Data Availability

The original contributions presented in this study are included in the article. Further inquiries can be directed to the corresponding author.
